# Acute Encephalopathy in a Patient With Chronic Isotretinoin Exposure and Frequent Rizatriptan Use: A Case Report

**DOI:** 10.1002/ccr3.73233

**Published:** 2026-07-25

**Authors:** Soheil Roshanzamiri, Mitra Rahimi, Shahin Shadnia, Peyman Erfantalab Evini, Faraz Zandiyeh, Babak Mostafazadeh

**Affiliations:** ^1^ Toxicological Research Center, Excellence Center & Department of Clinical Toxicology, School of Medicine Shahid Beheshti University of Medical Sciences Tehran Iran

**Keywords:** case report, encephalopathy, intracranial hypertension, isotretinoin, neurotoxicity, rizatriptan

## Abstract

Acute encephalopathy in patients with prolonged isotretinoin exposure should prompt careful assessment of undisclosed or non‐prescribed medication use. This case suggests a possible association between high cumulative isotretinoin exposure, frequent daily rizatriptan use, and acute encephalopathy, but does not establish a definitive drug–drug interaction, requiring a cautious assessment of causality.

## 1 Introduction

Isotretinoin is an effective systemic retinoid recommended for severe, recalcitrant, or treatment‐resistant acne vulgaris. Current acne guidelines continue to include isotretinoin among the recommended systemic options for eligible patients; however, its use requires careful monitoring due to teratogenic, mucocutaneous, metabolic, hepatic, musculoskeletal, and potential neuropsychiatric adverse effects [1, 2].

Neurologic complications associated with isotretinoin are uncommon but clinically important. Reported events include headache, mood and behavioral alterations, intracranial hypertension, and rare cases of encephalopathy [3, 4, 5, 6]. Isotretinoin is lipophilic and can cross the blood–brain barrier. Both experimental and clinical literature indicate that retinoid signaling may affect hippocampal neurogenesis, oxidative stress, dopaminergic pathways, and serotonergic neurotransmission, thereby providing biological plausibility for central nervous system (CNS) effects in susceptible individuals [7, 8, 9, 10]. Rizatriptan is a selective serotonin 5‐hydroxytryptamine 1B/1D (5‐HT1B/1D) receptor agonist utilized as an abortive therapy for migraine attacks. It is intended for intermittent, as‐needed use rather than scheduled daily therapy. Although triptans have historically been discussed in relation to serotonin toxicity, expert reviews and population‐based data indicate that serotonin syndrome from triptans, particularly without serotonergic antidepressants or monoamine oxidase inhibitors, is rare and often overdiagnosed [11, 12].

We report a case of acute encephalopathy with markedly elevated cerebrospinal fluid opening pressure in a patient with prolonged, high cumulative isotretinoin exposure and frequent daily rizatriptan use. To our knowledge, this specific clinical association has not been previously documented. We present it as a possible or probable association, not as definitive evidence of a drug–drug interaction.

## 2 Case History and Examination

A 28‐year‐old Iranian man presented to the Poisoning Emergency Department of Loghman Hakim Hospital with acute‐onset dizziness, confusion, severe agitation, and altered mental status beginning approximately 6 h before admission. He had a history of nodulocystic acne and chronic migraine. There was no documented history of psychiatric illness, seizure disorder, head trauma, diabetes mellitus, hypertension, chronic liver disease, chronic kidney disease, or known autoimmune disease. Family members denied known illicit substance use or recent medication changes at presentation.

The patient weighed 83 kg, was 184 cm tall, and had a body mass index of 24.5 kg/m^2^. He had a smoking history of approximately 4 pack‐years and reported occasional, non‐regular alcohol use.

Medication history included isotretinoin 20 mg every 12 h, corresponding to 40 mg/day or approximately 0.48 mg/kg/day, taken continuously for nearly 3 years. The estimated cumulative exposure was approximately 520–530 mg/kg, substantially higher than conventional cumulative dosing targets in acne therapy (120–150 mg/kg). The patient and family reported that isotretinoin use had been unsupervised and arbitrary, without physician follow‐up, planned discontinuation, liver function monitoring, or lipid profile monitoring during this period.

Three weeks before admission, rizatriptan 10 mg had been prescribed on a pro re nata (PRN; as‐needed) basis for migraine attacks. Contrary to the intended PRN regimen, the patient reported taking two 10‐mg tablets daily (20 mg/day) for approximately three consecutive weeks. On the day of symptom onset, he had taken the same daily amount and denied taking a higher dose. The exact time of the last rizatriptan dose before symptom onset could not be reliably determined.

Upon admission, the Glasgow Coma Scale score was 14 out of 15. Vital signs indicated a temperature of 38.5°C, a heart rate of 115 beats per minute, a respiratory rate of 16 breaths per minute, blood pressure measured at 115/75 mmHg, and an oxygen saturation of 95% on room air. The patient presented with confusion, restlessness, pronounced irritability, and severe agitation, corresponding to a Richmond Agitation‐Sedation Scale (RASS) score of +3. He exhibited disorganized thoughts, frequent transitions between sitting and standing positions, hand clenching, repeated complaints, and loud verbal outbursts.

The neurologic examination revealed bilateral mydriasis with pupils approximately 5 mm in diameter and sluggish pupillary light reflexes. Deep tendon reflexes appeared normal. There was no evidence of muscle rigidity, spontaneous or inducible clonus, ocular clonus, hyperreflexia, focal neurologic deficits, seizure activity, or meningismus. Peripheral or central antimuscarinic features were not evident: symptoms such as dry mouth, anhidrosis, urinary retention, carphologia, or trailing‐off speech were absent. Mild flushing was observed and was attributed to agitation and excessive motor activity.

## 3 Differential Diagnosis, Investigations, and Treatment

The initial differential diagnosis included infectious meningoencephalitis, toxic‐metabolic encephalopathy, isolated isotretinoin‐related toxicity, atypical serotonin toxicity, antimuscarinic toxicity, sympathomimetic intoxication, autoimmune encephalitis, seizure‐related encephalopathy, thyroid dysfunction, and intracranial hypertension associated with retinoid exposure.

Laboratory investigations indicated leukocytosis, with a white blood cell count of 14.7 × 10^9^/L and a neutrophil predominance of 86.7%. Elevated lactate dehydrogenase was observed at 657 U/L, while creatine phosphokinase was mildly elevated at 299 U/L. Serum creatinine was transiently increased but normalized following aggressive intravenous fluid resuscitation. Hepatic transaminases, liver function tests, and lipid profile remained within normal limits upon admission. Venous blood gas analysis revealed mild respiratory alkalosis, indicated by a pH of 7.43 and a partial pressure of carbon dioxide (PCO_2_) of 31.7 mmHg, consistent with hyperventilation during episodes of agitation.

Thyroid function tests were within normal limits, including thyroid‐stimulating hormone (TSH) 1.8 mIU/L and free thyroxine (FT4) 1.2 ng/dL; the reported triiodothyronine (T3) value was also within the laboratory reference range.

A qualitative urine immunoassay toxicology screen yielded negative results for amphetamines, methadone, opiates, benzodiazepines, cocaine, and cannabinoids. Additionally, blood levels of phenytoin, phenobarbital, carbamazepine, lithium, sodium valproate, acetaminophen, and salicylate were not detected. Since confirmatory chromatography or mass spectrometry was not performed, exposure to substances outside the screening panel cannot be completely excluded.

Non‐contrast brain computed tomography (CT) showed no acute abnormality. Electroencephalography (EEG) showed generalized background slowing, consistent with diffuse encephalopathy, without epileptiform discharges. Lumbar puncture revealed a markedly elevated cerebrospinal fluid opening pressure of 60 cm H_2_O. Cerebrospinal fluid analysis showed no evidence of inflammation, with glucose 66 mg/dL, protein 31 mg/dL, white blood cell count 2 cells per microliter, and lactate 35 mg/dL. Bacterial culture of the cerebrospinal fluid was sterile, and polymerase chain reaction (PCR) testing for herpes simplex virus (HSV) was negative.

Brain magnetic resonance imaging (MRI) was not performed during hospitalization because the patient was intubated and MRI‐compatible ventilatory support was not available. This represents an important limitation because MRI could have helped exclude subtle inflammatory, vascular, structural, or posterior reversible encephalopathy‐related findings.

Infectious meningoencephalitis was considered unlikely given the absence of meningismus, noninflammatory cerebrospinal fluid, sterile cultures, and a negative HSV PCR. Serotonin toxicity was considered because of rizatriptan exposure, agitation, mydriasis, and fever; however, the patient did not meet Hunter Serotonin Toxicity Criteria because clonus and hyperreflexia were absent. Antimuscarinic toxicity was considered, but was less likely because characteristic peripheral and central signs were absent. Autoimmune encephalitis was not fully excluded by antibody testing; however, the abrupt onset, negative initial studies, rapid improvement without immunotherapy, and full recovery after medication discontinuation and supportive care made it less likely. The final working diagnosis was acute toxic encephalopathy with markedly elevated intracranial pressure, possibly associated with prolonged high cumulative isotretinoin exposure and frequent daily rizatriptan use.

Both isotretinoin and rizatriptan were permanently discontinued. Initial control of agitation was attempted with intravenous diazepam, with a cumulative dose of 70 mg in the emergency department. Because agitation remained refractory and could not be safely controlled, endotracheal intubation was performed for airway protection, safe sedation, and prevention of harm. The patient was admitted to the intensive care unit and received supportive care, intravenous hydration, and continuous midazolam infusion at 5–10 mg/h.

## 4 Outcome and Follow‐Up

The patient gradually improved during intensive care. He was successfully extubated on hospital day 5. After regaining full consciousness, he confirmed the history of unsupervised long‐term isotretinoin use and frequent daily rizatriptan consumption contrary to the prescribed PRN instruction.

He was discharged with instructions to avoid reexposure to both isotretinoin and rizatriptan. At 3‐month follow‐up, he had complete neurological recovery without recurrence of agitation, confusion, mydriasis, seizure‐like activity, or other neurologic symptoms. Isotretinoin and rizatriptan were not reintroduced. Acne was managed with topical therapy, and migraine attacks were treated with simple analgesics, including nonsteroidal anti‐inflammatory drugs. A brief period of low mood was reported after discharge, but no persistent psychiatric or neurological sequelae were documented.

## 5 Discussion

This case describes acute encephalopathy with severe agitation, diffuse EEG slowing, and markedly elevated cerebrospinal fluid opening pressure in a patient with prolonged high cumulative isotretinoin exposure and frequent daily rizatriptan use. The case is clinically relevant because the key medication history was initially undisclosed, the cumulative isotretinoin exposure was substantially above conventional acne treatment targets, and the patient used rizatriptan daily despite PRN prescribing.

Causality must be interpreted cautiously. Rizatriptan had been used for approximately 3 weeks before presentation, and the patient did not report a higher dose on the day symptoms began. This weakens any claim of an immediate temporal reaction. Therefore, the clinical course suggests a possible or probable association rather than a definitive drug–drug interaction. The hypothesis is that repeated daily rizatriptan exposure may have acted as a precipitating or permissive factor in a patient whose central nervous system had already been exposed to prolonged high cumulative retinoid exposure. This remains speculative and cannot be proven from a single case.

Isotretinoin‐associated encephalopathy is rare. More commonly discussed central nervous system concerns include headache, mood changes, depression, psychosis, and intracranial hypertension [3, 4, 5, 6]. The markedly elevated opening pressure in our patient is particularly relevant because isotretinoin and other vitamin A derivatives have been linked to intracranial hypertension. In this case, normal cerebrospinal fluid composition, negative infectious studies, and full recovery after discontinuation of the suspected medications support a toxic or drug‐associated process rather than infectious meningoencephalitis.

The relationship between triptans and serotonin toxicity also requires caution. Rizatriptan is a selective 5‐HT1B/1D receptor agonist and is not generally expected to cause life‐threatening serotonin toxicity in isolation. The American Headache Society position paper and subsequent population‐based evidence suggest that serotonin syndrome associated with triptan use, particularly without selective serotonin reuptake inhibitor (SSRI), serotonin‐norepinephrine reuptake inhibitor (SNRI), or monoamine oxidase inhibitor (MAOI) co‐exposure, is uncommon [11, 12]. Accordingly, we do not classify this case as classic serotonin syndrome. The absence of clonus, hyperreflexia, and rigidity argues against Hunter‐defined serotonin toxicity [13].

A more plausible interpretation is acute toxic encephalopathy with raised intracranial pressure occurring in the setting of prolonged retinoid exposure and frequent centrally acting antimigraine medication use. Experimental data suggest that retinoids can influence neurogenesis, oxidative stress, and monoaminergic signaling, including serotonergic pathways [7, 8, 9, 10, 14, 15]. Additional isotretinoin‐specific experimental evidence suggests that 13‐cis‐retinoic acid can alter intracellular serotonin levels and increase 5‐HT1A receptor and serotonin transporter levels in vitro, supporting the biological plausibility of retinoid‐related serotonergic modulation [16]. These mechanisms provide biological plausibility but do not establish causality.

Compared with previously reported cases of isotretinoin‐associated encephalopathy, the present case is notable for unusually prolonged exposure, a very high cumulative dose of isotretinoin, markedly elevated cerebrospinal fluid opening pressure, and concurrent frequent daily rizatriptan use. Compared with triptan‐related serotonin toxicity reports, the present case lacked the neuromuscular findings required for Hunter‐defined serotonin toxicity and occurred without concomitant SSRI, SNRI, MAOI, linezolid, tramadol, or other strongly serotonergic co‐exposure. This distinction is important because overstating serotonin syndrome would be inconsistent with the clinical findings and the broader triptan safety literature. Although pharmacokinetic and pharmacodynamic interactions with triptans have been reviewed [17], no previously documented interaction between isotretinoin and rizatriptan was identified.

The Naranjo Adverse Drug Reaction Probability Scale [18] was applied to structure causality assessment. Points were assigned as follows: event occurring after exposure to the suspected drugs (+2), improvement after discontinuation and supportive care (+1), absence of a more likely alternative explanation after infectious, toxicologic, metabolic, EEG, CT, thyroid, and cerebrospinal fluid evaluation (+2), and objective evidence of encephalopathy/elevated intracranial pressure through EEG and lumbar puncture (+1). No points were assigned for rechallenge, drug‐level confirmation, prior conclusive reports of this specific interaction, or dose escalation immediately before presentation. The total score was 6, consistent with a probable adverse drug reaction.

This case has several limitations. First, it is a single case, and causality remains circumstantial. Second, the exact clock time of the last rizatriptan dose before symptom onset could not be reliably established. Third, an MRI could not be obtained because the patient was intubated and MRI‐compatible ventilation was unavailable. Fourth, the toxicology panel was based on qualitative urine immunoassay and selected blood drug levels rather than confirmatory chromatography or mass spectrometry, so substances outside the tested panel cannot be completely excluded. Fifth, antibody testing for autoimmune encephalitis was not performed. Sixth, fundoscopic findings and serial cerebrospinal fluid pressure measurements were unavailable, limiting interpretation of the markedly elevated opening pressure. Finally, intentional rechallenge was neither ethical nor clinically appropriate.

This case highlights acute encephalopathy with markedly elevated cerebrospinal fluid opening pressure in a patient with prolonged high cumulative isotretinoin exposure and frequent daily rizatriptan use. The findings suggest a possible or probable association rather than a definitive drug–drug interaction. Clinicians should carefully investigate unsupervised medication use, cumulative retinoid exposure, and non‐prescribed dosing patterns when evaluating acute agitation or altered mental status. These limitations should be considered when interpreting causality.

## Author Contributions

**Soheil Roshanzamiri:** conceptualization, methodology, writing – original draft, writing – review and editing. **Mitra Rahimi:** conceptualization, methodology, resources. **Shahin Shadnia:** supervision. **Peyman Erfantalab Evini:** conceptualization, investigation, resources. **Faraz Zandiyeh:** investigation, methodology. **Babak Mostafazadeh:** resources, supervision, validation.

## Funding

The authors have nothing to report.

## Consent

Written informed consent was obtained from the patient for publication of this case report in accordance with the journal's patient consent policy.

## Conflicts of Interest

The authors declare no conflicts of interest.

## Data Availability

Deidentified data supporting the findings of this case report are available from the corresponding author upon reasonable request and subject to patient confidentiality and institutional restrictions.

## References

[1] R. V. Reynolds , H. Yeung , C. E. Cheng , et al., “Guidelines of Care for the Management of Acne Vulgaris,” Journal of the American Academy of Dermatology 90, no. 5 (2024): 1006.e1–1006.e30. 10.1016/j.jaad.2023.12.017 38300170

[2] A. L. Zaenglein , A. L. Pathy , B. J. Schlosser , et al., “Guidelines of Care for the Management of Acne Vulgaris,” Journal of the American Academy of Dermatology 74, no. 5 (2016): 945–973. 26897386 10.1016/j.jaad.2015.12.037

[3] J. E. Strahan and S. Raimer , “Isotretinoin and the Controversy of Psychiatric Adverse Effects,” International Journal of Dermatology 45, no. 7 (2006): 789–799. 16863513 10.1111/j.1365-4632.2006.02660.x

[4] D. I. Friedman , “Medication‐Induced Intracranial Hypertension in Dermatology,” American Journal of Clinical Dermatology 6, no. 1 (2005): 29–37. 15675888 10.2165/00128071-200506010-00004

[5] A. Wong , M. Williams , and W. Gibb , “Isotretinoin‐Induced Encephalopathy,” Journal of Dermatological Treatment 21, no. 6 (2010): 361–362. 20059367 10.3109/09546630903341978

[6] L. Le Guennec , C. Marois , S. Demeret , E. F. M. Wijdicks , and N. Weiss , “Toxic‐Metabolic Encephalopathy in Adults: Critical Discussion and Pragmatical Diagnostic Approach,” Revue Neurologique (Paris) 178, no. 1–2 (2022): 93–104. 10.1016/j.neurol.2021.11.007 34996631

[7] Y. D. Fragoso , K. D. Shearer , A. Sementilli , L. V. de Carvalho , and P. J. McCaffery , “High Expression of Retinoic Acid Receptors and Synthetic Enzymes in the Human Hippocampus,” Brain Structure & Function 217, no. 2 (2012): 473–483. 22075950 10.1007/s00429-011-0359-0 PMC3322324

[8] J. Crandall , Y. Sakai , J. Zhang , et al., “13‐Cis‐Retinoic Acid Suppresses Hippocampal Cell Division and Hippocampal‐Dependent Learning in Mice,” Proceedings of the National Academy of Sciences of the United States of America 101, no. 14 (2004): 5111–5116. 15051884 10.1073/pnas.0306336101 PMC387382

[9] K. O'Reilly , S. J. Bailey , and M. A. Lane , “Retinoid‐Mediated Regulation of Mood: Possible Cellular Mechanisms,” Experimental Biology and Medicine 233, no. 3 (2008): 251–258. 18296731 10.3181/0706-MR-158

[10] J. D. Bremner , N. Fani , A. Ashraf , et al., “Functional Brain Imaging Alterations in Acne Patients Treated With Isotretinoin,” American Journal of Psychiatry 162, no. 5 (2005): 983–991. 15863802 10.1176/appi.ajp.162.5.983

[11] R. W. Evans , S. J. Tepper , R. E. Shapiro , C. Sun‐Edelstein , and G. E. Tietjen , “The FDA Alert on Serotonin Syndrome With Use of Triptans Combined With Selective Serotonin Reuptake Inhibitors or Selective Serotonin‐Norepinephrine Reuptake Inhibitors: American Headache Society Position Paper,” Headache 50, no. 6 (2010): 1089–1099. 20618823 10.1111/j.1526-4610.2010.01691.x

[12] Y. Orlova , P. Rizzoli , and E. Loder , “Association of Coprescription of Triptan Antimigraine Drugs and Selective Serotonin Reuptake Inhibitor or Selective Norepinephrine Reuptake Inhibitor Antidepressants With Serotonin Syndrome,” JAMA Neurology 75, no. 5 (2018): 566–572. 29482205 10.1001/jamaneurol.2017.5144 PMC5885255

[13] E. J. C. Dunkley , G. K. Isbister , D. Sibbritt , A. H. Dawson , and I. M. Whyte , “The Hunter Serotonin Toxicity Criteria: Simple and Accurate Diagnostic Decision Rules for Serotonin Toxicity,” QJM 96, no. 9 (2003): 635–642. 12925718 10.1093/qjmed/hcg109

[14] Y. Alabdaly , S. Abdul Hameed , and N. Ibrahim , “Neurotoxicity of Isotretinoin in Mice: Behavioral and Tissue Neurological Function Assessment,” Iranian Journal of Veterinary Medicine 19, no. 4 (2025): 685–694, 10.32598/IJVM.19.4.1005588.

[15] B. C. Melnik , “Apoptosis May Explain the Pharmacological Mode of Action and Adverse Effects of Isotretinoin, Including Teratogenicity,” Acta Dermato‐Venereologica 97, no. 2 (2017): 173–181. 27671426 10.2340/00015555-2535

[16] K. C. O'Reilly , S. Trent , S. J. Bailey , and M. A. Lane , “13‐Cis‐Retinoic Acid Alters Intracellular Serotonin, Increases 5‐HT1A Receptor, and Serotonin Reuptake Transporter Levels In Vitro,” Experimental Biology and Medicine 232, no. 9 (2007): 1195–1203, 10.3181/0703-RM-83. 17895527

[17] P. E. Rolan , “Drug Interactions With Triptans,” CNS Drugs 26, no. 11 (2012): 949–957. 23018546 10.1007/s40263-012-0002-5

[18] C. A. Naranjo , U. Busto , E. M. Sellers , et al., “A Method for Estimating the Probability of Adverse Drug Reactions,” Clinical Pharmacology and Therapeutics 30, no. 2 (1981): 239–245. 7249508 10.1038/clpt.1981.154

